# A Clearer Picture: Using Fetal MRI to Diagnose Neck Masses and Predict Airway Compromise

**DOI:** 10.1002/pd.70078

**Published:** 2026-01-24

**Authors:** Louise Wilson, Elspeth H. Whitby, Ashok Raghavan

**Affiliations:** ^1^ Department of Clinical Sciences School of Medicine and Population Health University of Sheffield Sheffield UK; ^2^ Medical Imaging and Medical Physics Sheffield Teaching Hospitals Sheffield UK; ^3^ Department of Radiology Sheffield Children's Hospital Sheffield UK

## Abstract

**Introduction:**

Fetal neck masses are rare but can be life‐threatening if causing airway compromise. Early and accurate diagnosis of these masses allows life‐saving interventions to be undertaken at birth in the form of the EXIT procedure.

**Methods:**

A single institution case series of all patients referred for fetal MRI to a tertiary center in the North of England due to presence of a neck mass on antenatal ultrasound. Data concerning the MRI findings for each patient and their final diagnosis were collected to create a flow chart proposing the most likely diagnosis based on fetal MRI features.

**Results:**

13 patients who underwent fetal MRI for a neck mass with a final diagnosis available were included in the analysis. This review shows the range of diagnoses in these patients and that MRI was accurate in predicting airway compression and the need for the EXIT procedure.

**Conclusion:**

Fetal MRI is a valuable tool in addition to ultrasound for refining the diagnosis of masses of the fetal neck and assessment of airway patency to allow planning for management at birth.

## Summary

- What's already known about this topic?
  ◦ Early diagnosis of fetal neck masses is important for antenatal counselling and for planning management at birth.
- What does this study add?
  ◦ Use of fetal MRI allows refinement of the diagnosis and accurate assessment of airway patency prior to birth.

**Abbreviations**
BMI: Body Mass Index
DWI: Diffusion Weighted Imaging
EXIT: Ex Utero Intrapartum Treatment
ISSVA: International Society for the Study of Vascular Anomalies
MDT: Multidisciplinary Team
MRI: Magnetic Resonance Imaging
NHS: National Health Service
SSFSE: Single Shot Fast Spin Echo

## 1 Introduction

There are several different pathologies which present as masses in the fetal neck. The most common include lymphatic malformations and teratomas as well as less frequently seen pathologies such as malignant tumors including rhabdomyosarcomas, and goiters caused by maternal thyroid disease [1, 2] or certain medications or rare genetic causes [2, 3].

Cervical lymphatic malformations are the most common type of lymphatic malformation seen in infants, affecting 1.2–2.8/1000 live births [4]. They are low‐flow vascular malformations that do not communicate with normal lymphatic vessels. Head and neck malformations account for 70%–80% of all lymphatic malformations [5]. The International Society for the Study of Vascular Anomalies (ISSVA) updated the nomenclature for such malformations in 2018 and, in the new classification, the terms “lymphangioma” and “cystic hygroma” have been replaced with “lymphatic malformation” and “cavernous haemangioma” have been replaced by “venous malformation”. This is due to potential confusion with the older terms as the suffix “oma” implies a tumor rather than a malformation [4, 5].

Teratomas are congenital tumors containing tissue from all three germ cell layers (the mesoderm, ectoderm and endoderm) [6]. Cervical teratomas are rare and account for 3%–5% of all teratomas with an incidence of 1 in 20,000–1 in 40,000 live births [7], with the most common sites for fetal teratoma being in the sacrococcygeal region. In the head and neck, teratomas are usually benign [8].

Early and accurate diagnosis of fetal neck masses is important as it allows appropriate antenatal counselling for families and planning for management at delivery and in the postnatal period. Ultrasound remains the imaging modality of choice for screening for fetal anomalies. Magnetic resonance imaging (MRI) is essential for surgical planning, which can reduce maternal and fetal comorbidities and can be a useful adjunct in refining the diagnosis.

Fetal MRI has been shown to be highly accurate in the diagnosis of mass nature and assessment of the fetal airway, essential for planning of management at the time of delivery to improve maternal and fetal safety [9]. Whilst other studies have shown the diagnostic accuracy of fetal MRI in diagnosis of lymphatic and venous malformations to be no better than ultrasound they can be a useful adjunct in refining the diagnosis and highlight the complementary role of MRI in providing more anatomical detail to anticipate the required intervention for example the need for an EXIT delivery, and risk of complications for example issues with intubation, or breathing problems due to poor lung development at birth [10, 11].

The primary role of fetal MRI in clinical practice for assessment of neck mass is to assess for airway patency and to help refine a diagnosis in cases where ultrasound is technically challenging, such as high maternal body mass index (BMI) or unfavorable fetal position [12]. In cases with significant airway compromise, multi‐disciplinary team (MDT) planning involving the obstetric team for the delivery, the neonatal team for immediate post‐delivery care planning, the anesthetic team, the ear nose and throat specialists if intubation is likely to be required and problematic and the radiology team. The MDT is required prior to delivery to prepare for advanced airway intervention at birth [13]. For the most severe cases, where the airway is not patent, this intervention is the ex utero intrapartum treatment (EXIT) procedure. The EXIT procedure involves securing the airway via endotracheal intubation or tracheostomy in a controlled manner whilst the fetus is only partially delivered and remains attached to the placenta [14]. EXIT procedures are relatively uncommon, require an experienced MDT in a tertiary center and are associated with both maternal (e.g., hemorrhage, infection) and fetal risks (e.g., injury, hypoxia) [15].

The aim of this study was to present a series of patients with fetal neck masses as a pictorial review of the different diagnoses. Secondary aims were to develop a pathway of the most likely diagnosis based on MRI findings and to assess the accuracy of MRI to depict airway patency.

## 2 Methods

This study was a retrospective case series from a single institution. Ethical approval was given by the Health Research Authority (HRA) for retrospective data collection and analysis (IRAS project ID 222053). The study included all patients referred to our center for fetal MRI with a neck mass from January 2011 to March 2023. Oral lesions and encephaloceles were excluded.

The MRI scans were performed using a 1.5 T S Avanto scanner (Erlangen, Germany). The assessment of the neck mass and surrounding anatomical structures was made using the Agfa Healthcare (Mortsel, Belgium) Enterprise Imaging platform. The assessments were made predominantly from the T2‐weighted images, with the diffusion weighted imaging (DWI) sequences used to assess for any restricted diffusion within the masses. The MRI findings were independently reported by two consultant radiologists with over 20 years' experience of fetal MRI blinded to the outcome and each other. Each radiologist reported the size of the neck mass in terms of anterior‐posterior (AP), medio‐lateral (ML) and cranio‐caudal (CC) dimensions. They also commented on airway patency, location of the mass within the neck, the nature of the mass that is heterogeneity and whether it was solid or cystic, presence of restriction on diffusion weighted imaging and the most likely diagnosis. A weighted Cohen's Kappa was then obtained for these data using IBM SPSS Statistics for Macintosh version 29.0.1.0 to determine the degree of inter‐observer reliability. In case of disagreement a face to face discussion to reach consensus was planned.

Data were collected concerning the ultrasound findings prompting referral for MRI, gestation at the time of MRI scan and the fetal MRI findings. Patient outcomes, including the outcome of the pregnancy and the final histological diagnosis from surgical biopsy or postmortem, were collected. Additional information concerning whether an EXIT procedure was undertaken and the type of airway and respiratory support needed after birth were also examined in detail. Where patients had been referred from external sites, these centers were contacted directly for this information. The diagnosis, as written in the patient notes, was documented in the analysis, alongside the most up‐to‐date terminology as listed in the ISSVA classification [16].

A flow chart detailing the most likely diagnoses based on the fetal MRI findings was then developed using data from the MRI reports and the final diagnosis for the patients. This was created independently by each consultant radiologist and the charts were then combined following discussion. The main MRI findings used to develop the flow chart were the nature of the mass, i.e. whether cystic or solid, the location of the mass within the neck, its heterogeneity and the presence or absence of restriction with DWI.

## 3 Results

Twenty patients met the inclusion criteria of having undergone fetal MRI at our center due to the presence of a neck mass on antenatal ultrasound. Seven patients had no final diagnosis, two had no outcome data available, three underwent termination of pregnancy without postmortem examination, and two were stillborn but did not undergo postmortem examination.

**FIGURE 1:**
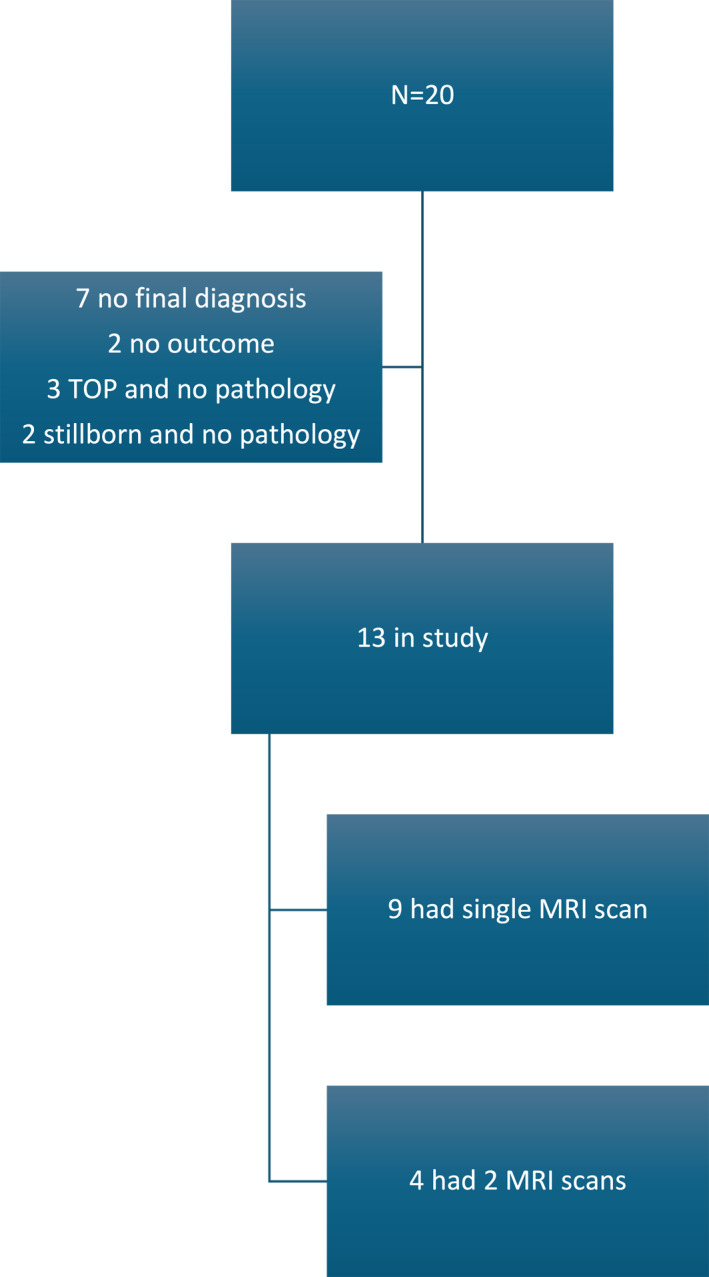
Flow chart of the patient group. TOP, termination of pregnancy; MRI Magnetic, resonance scan.

A final diagnosis was available for 13 patients, all included in the final analysis (Figure 1).

The mean gestational age at the time of fetal MRI was 28 weeks and 3 days with a range of 21–36 weeks. Of the 13 patients included in the final analysis, five were referred for fetal MRI from external tertiary centers. Four patients underwent two fetal MRI scans during pregnancy; the MRI findings listed below for these patients are from the first MRI scan. The first scan was chosen for consistency and in all cases the management plan had been based on the initial scan. All EXIT cases had only one scan as it was considered sufficient for management and all initial scans were between 31 weeks GA and 33 weeks GA. Second scans were performed when the first scan was 22 weeks GA or earlier; this included a case where an EXIT was deemed impossible on the first scan and to ensure this remained the same, for the fetal goiter to assess growth and airway compression, and for teratoma and hemangiolymphoma. One baby was born prior to the planned second scan.

### 3.1 Fetal MRI Findings

The fetal MRI findings for each patient alongside their final diagnosis are shown in Table 1. There were five cases with airway compression (*n* = 3) or displacement (*n* = 2) seen on the MRI.

**TABLE 1:** Fetal MRI findings and postnatal diagnosis for each patient.

Postnatal diagnosis	Gestation at MRI	Mean AP	Mean ML	Mean CC	Solid/cystic	Hetero/homogeneity	DWI	Airway patency
Rhabdomyosarcoma	33 weeks	41 mm	42 mm	33.5 mm	Solid	Homogeneous	Not done	Patent but displaced
Teratoma	22 weeks (repeat at 29 weeks)	50.5 mm	52 mm	51.5 mm	Mixed	Heterogeneous	Restriction in solid component	Patent but displaced
Kaposiform lymphangiomatosis (1)	27 weeks	29.5 mm	28 mm	34 mm	Cystic	Heterogeneous	No restriction	Patent
Teratoma	36 weeks	54.5 mm	64.5 mm	53 mm	Mixed	Heterogeneous	No restriction	Compressed
Lymphatic malformation	31 weeks	68.5 mm	37 mm	68.5 mm	Cystic	Heterogeneous	No restriction	Small compressed area
Lymphatic malformation	35 weeks	96 mm	51 mm	79.5 mm	Cystic	Heterogeneous	No restriction	Patent
Lymphatic malformation	21 weeks (repeat at 31 weeks)	27 mm	19 mm	21.5 mm	Cystic	Heterogeneous	No restriction	Patent
Macrocystic lymphatic malformation in posterior neck (documented as hygroma)	36 weeks	51.5 mm	39.5 mm	80.5 mm	Cystic	Homogeneous	No restriction	Patent
Kaposiform haemangio‐endothelioma (2)	22 weeks	7 mm	11 mm	7.5 mm	Cystic	Heterogeneous	No restriction	Patent
Lymphatic malformation	25 weeks	80 mm	79 mm	51 mm	Cystic	Homogeneous	No restriction	Patent
Teratoma	31 weeks (repeat at 34 weeks)	64 mm	69.5 mm	56 mm	Mixed	Heterogeneous	Restriction in solid component	Patent
Rhabdomyosarcoma	30 weeks	59 mm	56.5 mm	50.5 mm	Solid	Homogeneous	Restriction	Compressed
Goiter from thyroid dyshormonogenesis	21 weeks (repeat at 30 weeks)	9.5 mm	9.25 mm	18.25 mm	Solid	Homogeneous	No restriction	Patent

*Note:* The prenatal diagnosis was correct in all but 2 cases (1) Kaposiform lymphangiomatosis was thought to be a lymphangioma and (2) Kaposiform haemangio‐endothelioma was thought to be a haemangiolymphangioma. Light shading indicates a case with patent but displaced airways. Dark shading indicates a case with compressed airway. No sahding indicates a case with a patent ariway.

Abbreviations: AP, antero‐posterior; CC, cranio‐caudal; DWI, diffusion weighted imaging; ML, medio‐lateral.

The weighted Cohen's Kappa, which was undertaken to assess the degree of inter‐observer reliability, was 0.769, which showed good agreement between the two clinicians measuring the size of the neck masses from the MRI.

The observers had agreement on diffusion restriction, location and nature of the mass. There was disagreement on two cases over airway patency, where one reported displacement but patent and the other compressed. The final displaced but patent decision was agreed by consensus on image review. The second case was a patent versus a compressed, agreed as patent by consensus. Neither of these cases had an EXIT procedure.

### 3.2 Postnatal Outcomes and Diagnoses

All 13 patients were liveborn. The mean gestational age at delivery was 35 weeks and 5 days. Range 29 weeks and 1 day to 38 weeks and 3 days. Two patients died in the neonatal period. One of these was following a planned palliative delivery (large tumor with no access for an EXIT procedure and fetal hydrops) and the other from Kasabach‐Merritt phenomenon, which is a disorder of thrombocytopenia and hemorrhage secondary to a Kaposiform haemangioendothelioma, a locally aggressive rare vascular tumor. This was not predicted but was expected given the diagnosis. To our knowledge, the remaining patients are alive and well, having undergone treatment after birth or are still receiving treatment.

Four of the patients underwent the EXIT procedure at delivery, two of these patients required immediate tracheostomy and were cases with known airway compression on the MRI. One of the EXIT procedures was performed at our center, and the other three procedures were performed elsewhere. As predicted from the MDT discussion, the four patients who underwent planned EXIT procedures all had significant airway compromise or displacement seen on MRI as reported by both of the radiologists. The fifth patient with known airway compression was the patient who had planned palliative care (see above). One other patient required intubation after birth due to poor respiratory effort. They had a small area of airway compression noted on fetal MRI, but underwent a straightforward intubation by the neonatal team. None of the remaining eight patients required airway support or invasive ventilation after birth. One was electively intubated for a postnatal MRI scan and had previously required non‐invasive ventilation due to prematurity (born at 33 weeks). Two other patients briefly required low flow oxygen therapy.

The final diagnoses for these patients included lymphatic malformation (*n* = 5), of which one was documented as a cystic hygroma, teratoma (*n* = 3), rhabdomyosarcoma (*n* = 2), Kaposiform haemangioendothelioma (*n* = 1), Kaposiform lymphangiomatosis (*n* = 1) and goiter secondary to congenital hypothyroidism caused by thyroid dyshormonogenesis (*n* = 1). Rhabdomyosarcomas are rare malignant soft tissue tumors arising from the embryonal mesenchyme and require complex oncology management including surgery, chemotherapy and radiotherapy [17, 18]. Kaposiform haemangioendotheliomas are rare, locally aggressive vascular tumors with significant morbidity and mortality due to local invasive and compression as well as the consumptive coagulopathy Kasabach‐Merritt phenomenon [19]. Kaposiform lymphangiomatosis is a rare lymphatic anomaly characterized by abnormal lymphatic channels and clusters of lymphatic endothelial cells with a spindled or “kaposiform” morphology [20].

**FIGURE 2:**
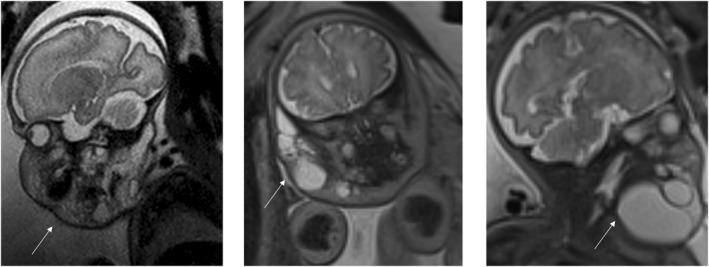
Fetal MRI images of lymphatic malformation. Left image ‐ T2 haste sagittal image of a 31 weeks fetus showing a multi‐septated cystic mass from orbit to upper thorax, which infiltrates into the face with airway compression. Middle image ‐ T2 haste coronal image of a 35 weeks fetus showing a septated multi‐cystic subcutaneous lesion right side of the face from orbit to neck. Right image ‐ T2 haste coronal image with head turned to the left of a 25 weeks fetus with an anterior cystic neck mass.

**FIGURE 3:**
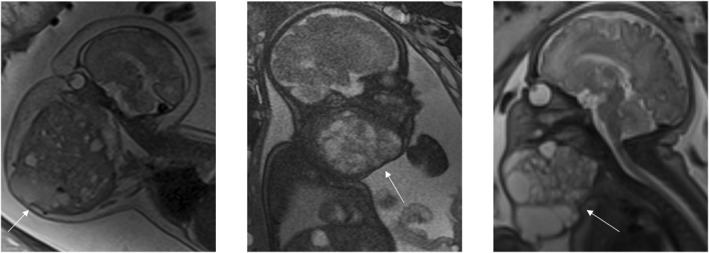
Fetal MRI images of teratoma. Left image ‐ T2 haste sagittal image of a 22 weeks fetus with an extensive neck and facial mass with solid and cystic components and airway displacement. Middle image ‐ FIESTA 60 sagittal imaging of fetus with a large cystic/solid mass on the left side of the neck, crossing over midline with Compression of the vessels and trachea. Right image ‐ T2 haste sagittal image of a 31 weeks fetus with an anterior cystic neck mass with some solid components.

**FIGURE 4:**
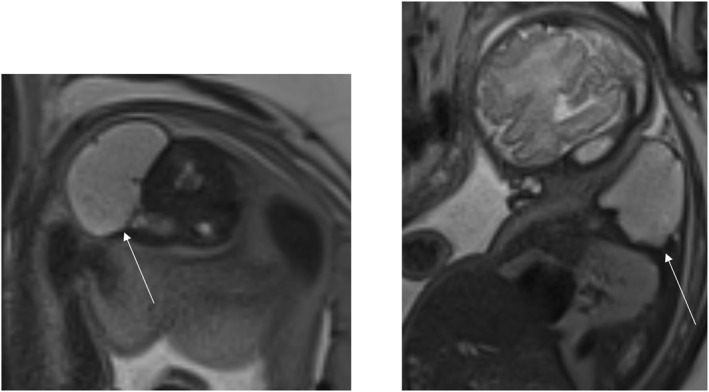
Fetal MRI images of a macrocystic lymphatic malformation (documented as cystic hygroma). T2 axial (left) and sagittal (right) images of a 36 weeks fetus with a right sided homogeneous macrocystic cystic neck mass.

**FIGURE 5:**
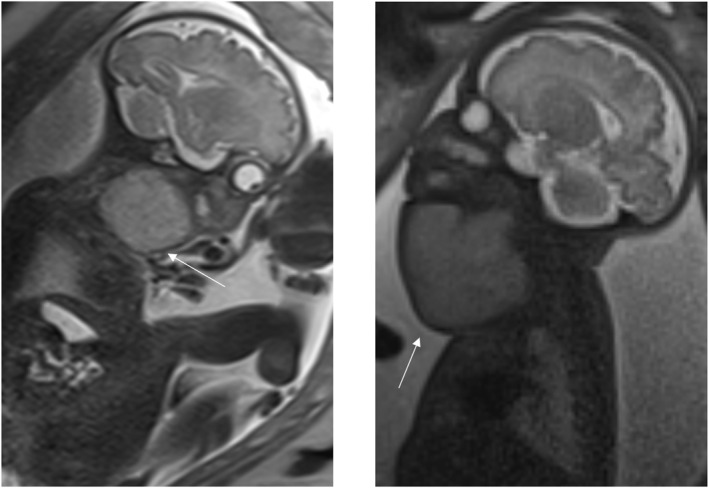
Fetal MRI images of rhabdomyosarcoma. T2 haste sagittal images of a 33 weeks fetus (left) and a 30 weeks fetus (right) both with a solid anterior neck mass causing airway compression/displacement.

**FIGURE 6:**
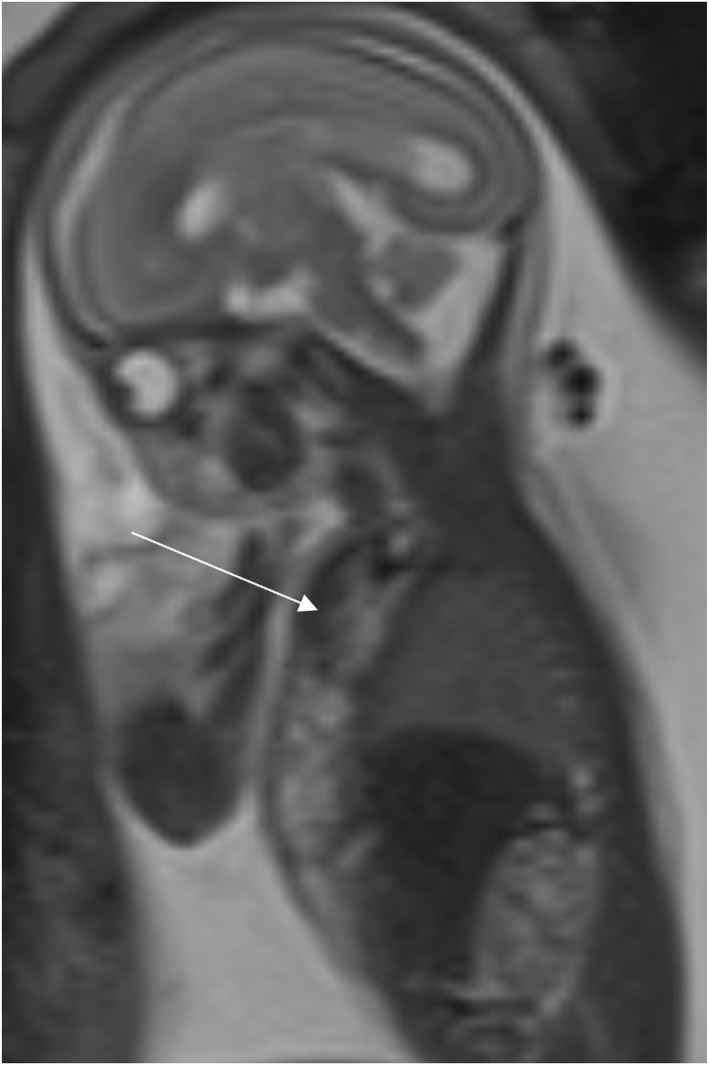
Fetal MRI image of a Kaposiform haemangioendothelioma. T2 haste sagittal image of a 22 weeks fetus with extensive subcutaneous mixed venous and lymphatic malformation and extension into the neck and anterior aspect of the mediastinum consistent with the known lymphatic drainage pattern.

**FIGURE 7:**
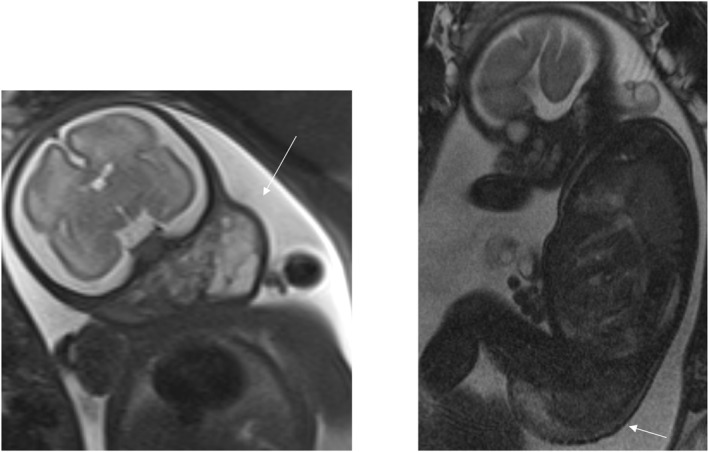
Fetal MRI images of Kaposiform lymphangiomatosis. Left image ‐ T2 haste coronal image of 27 weeks fetus with a multiloculated fluid filled cystic lesion that extends from the subcutaneous tissues on the left side of the neck anteriorly and posteriorly toward the midline. Extends from shoulder to ear with possible areas of hemorrhage. Right image ‐ T2 sagittal image of the same fetus showing a similar cystic lesion over the left buttock and thigh.

**FIGURE 8:**
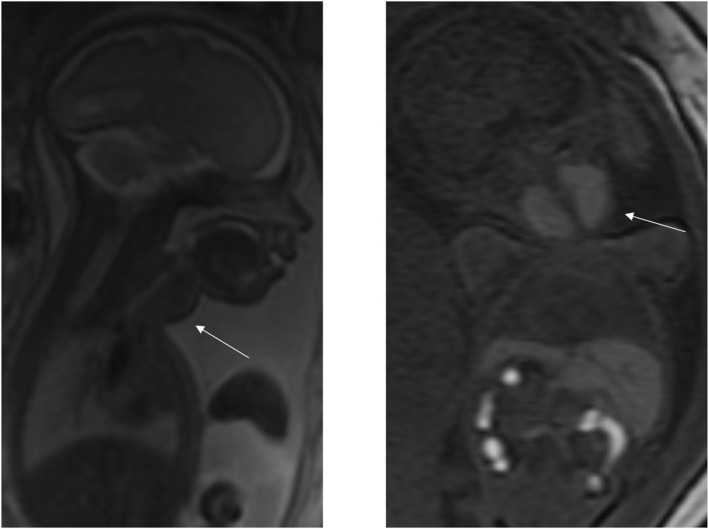
Fetal MRI images of a goiter. Left image ‐ T2 SSFSE sagittal image of 21 weeks fetus showing a solid homogeneous anterior neck mass. Right image ‐ T1 coronal image of the same fetus showing increased signal in the thyroid (goiter) and the meconium in the bowel.

The four patients who underwent the EXIT procedure had diagnoses of rhabdomyosarcoma (*n* = 2), lymphatic malformation (*n* = 1) and teratoma (*n* = 1). The fetal MRI images for each of the diagnoses are shown in the figures below (Figures 2, 3, 4, 5, 6, 7, 8).

### 3.3 Diagnostic Flow Chart

**FIGURE 9:**
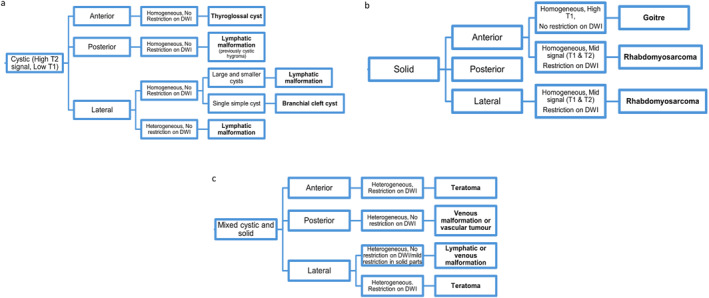
Flow chart of most likely diagnosis of neck mass seen based on fetal MRI findings. (a–c) Flow charts of most likely diagnosis of neck mass seen based on fetal MRI findings. (a) Cystic lesions. (b) Solid lesions. (c) Mixed cystic solid lesions.

The diagnostic flow chart was developed using the MRI findings from this cohort compared with the final diagnosis and other research reported in the literature. Figure 9a–c highlights the most likely diagnosis based on the MRI findings, including the nature of the neck mass, that is, whether cystic or solid, the site of the mass, and the heterogeneity and MRI signal uptake.

Whilst there was some overlap between the MRI findings for different diagnoses, the most common diagnoses had key defining features on MRI. Lymphatic malformations were cystic or mixed cystic and solid lesions, which could be homogeneous or heterogeneous, but there was no restriction seen on diffusion weighted imaging. Teratomas were mixed cystic and solid lesions with restriction on DWI. The patient recorded as having a cystic hygroma, now known as macrocystic lymphatic malformation, had a cystic lesion only seen in the posterior neck. Rhabdomyosarcomas were solid, homogeneous lesions with mid‐signal and restriction on DWI. We did not have any cases of congenital hemangiomas.

## 4 Discussion

This study presents the range of 13 neck masses referred for fetal MRI to a tertiary fetal and neonatal center in England over a 12‐year period. It showcases the different diagnoses seen including the more common lymphatic malformations and rarer conditions such as teratomas and vascular tumors and their appearances on MRI in utero. We used the MRI findings to propose a pathway indicating the most likely diagnosis although this has not been validated on any new cases and is suggested as an aid to diagnosis not a diagnostic tool.

This research highlights the role of fetal MRI in addition to antenatal ultrasound in these cases to refine the diagnosis and assess the patency of the airway. Accurate prenatal characterization prior to delivery enables antenatal planning for postnatal management, counselling of parents and multidisciplinary team (MDT) discussions to plan the birth, the teams required at birth and the postnatal care to reduce maternal and fetal co‐morbidities. Several of these cases were complex, with extensive lymphatic lesions and some with lesions at multiple sites. The fetal MRI was able to provide detailed additional information on the sites of lymphatic lesions and potential metastatic sites of malignant lesions and to look for other structural anomalies of the fetus which impact morbidity and mortality.

Mass size did not appear to help in characterization as reported previously [9, 10, 14], whilst location helps characterization [12, 13] and has been reported as the best indicator of likely airway compression [10, 14].

As shown in other studies [9, 14], a significant advantage of using fetal MRI in these cases was the accurate prediction of airway patency in advance of delivery. All our cases were referred for both assessment of the nature of the mass and assessment of the airways as ultrasound did not provide details on airway patency. This allowed complex MDT planning for management at birth, including EXIT procedures. The MRI prediction of airway patency remained accurate even when the MRI had been performed several weeks prior to delivery. In this cohort, the overall size of the mass did not relate to the presence of airway compression.

The study is limited by the relatively small cohort, retrospective design and the missing data concerning the final diagnosis for seven of the twenty patients initially identified from the MRI database. As this patient group underwent their MRI scans as part of their routine National Health Service (NHS) care, the MRI scans were all performed at different gestations, meaning measurements cannot be standardized. The MRI performed did not use the more advanced techniques of super resolution reconstruction or virtual reality/3D modeling, which could be utilized in the future to provide further additional information for the delivery process [21, 22].

## 5 Conclusion

In conclusion, this study shows the heterogeneity of neck masses diagnosed in utero and the role of fetal MRI in refining the diagnosis and predicting airway patency to guide management at birth.

## Funding

The authors have nothing to report.

## Ethics Statement

Ethical approval was given by the Health Research Authority (HRA) for retrospective data collection and analysis (IRAS project ID 222053). As this was an anonymized case series, specific patient consent was not collected from parents/guardians.

## Conflicts of Interest

The authors declare no conflicts of interest.

## Data Availability

The data that support the findings of this study are available from the corresponding author upon reasonable request.
